# Supplementary material for: Timed daily exercise remodels circadian rhythms in mice
Source: Commun Biol. 2021 Jun 18;4:761. doi: 10.1038/s42003-021-02239-2 (PMC8213798; doi:10.1038/s42003-021-02239-2)
Supplement: Supplementary file 2 — Supplementary Information [file 42003_2021_2239_MOESM2_ESM.pdf]

Supplementary materials to:

Hughes, Samuels, Baño-Otálora, et al. (2021) Timed daily exercise restores circadian rhythms in mice. **Communications Biology**.

Index:

Supplementary Figure 1: 2
Supplementary Figure 2: 4
Supplementary Figure 3: 5
Supplementary Figure 4: 6
Supplementary Figure 5: 7
Supplementary Table 1: 8
Supplementary Table 2: 9

Figure-S1

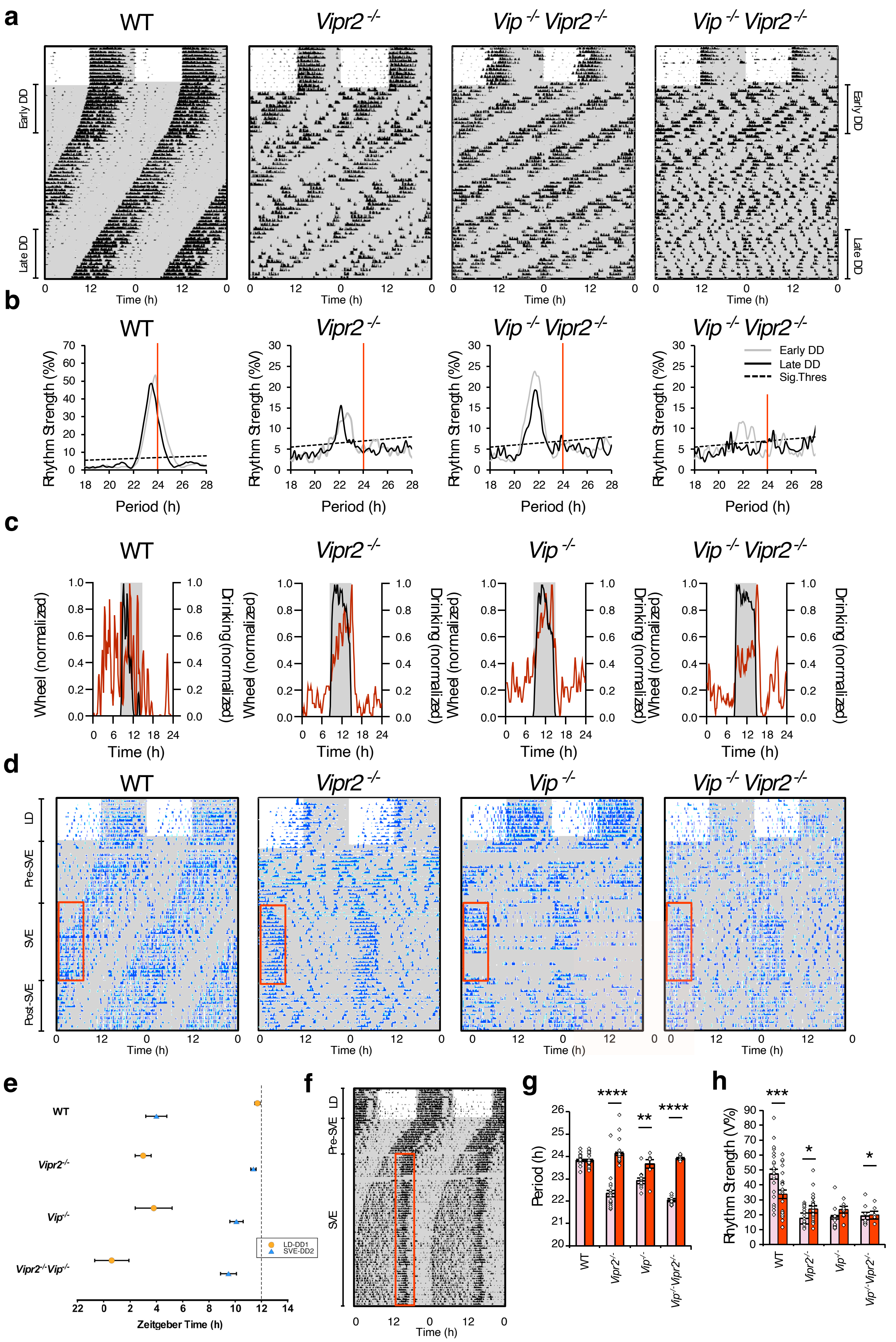
a
WT
Vipr2 -/-
Vip -/- Vipr2 -/-
Vip -/- Vipr2 -/-
Early DD
Late DD
Time (h)
b
Rhythm Strength (%V)
Period (h)
Early DD
Late DD
Sig.Thres
c
Vip -/-
Wheel (normalized)
Drinking (normalized)
Time (h)
d
LD
Pre-SVE
SVE
Post-SVE
Time (h)
e
WT
Vipr2-/-
Vip-/-
Vipr2-/-Vip-/-
LD-DD1
SVE-DD2
Zeitgeber Time (h)
f
LD
Pre-SVE
SVE
Time (h)
g
Period (h)
WT
Vipr2-/-
Vip-/-
Vip-/-Vipr2-/-
****
**
****
h
Rhythm Strength (V%)
***
*
*

Figure S1.

(**a-b**) **Mice with disrupted VIP-VPAC$_2$ signaling do not spontaneously generate robust ~24h rhythms in wheel-running activity.** (**a**) Representative double-plotted actograms showing locomotor wheel-running activity for WT (C57BL/6), *Vipr2$^{-/-}$* and *Vip$^{-/-}$Vipr2$^{-/-}$* mice (n=6, n=6 and n=9, respectively) maintained in DD with *ad libitum* running wheel availability (i.e. without SVE) for durations similar to 3-week SVE experiments (~8 weeks in DD in total). Shaded areas represent darkness. (**b**) *Chi^2* periodograms showing dominant circadian period of running wheel activity in both early DD (gray; equivalent to pre-SVE in **Fig. 1**) and late DD (black; equivalent to post-SVE, in **Fig. 1**). Diagonal broken lines indicate significance threshold at p=0.0001. Vertical red lines indicate 24h period for visual reference relative to peak behavioral period at early and late DD.

(**c-d**) **Mice with disrupted VIP-VPAC$_2$ signaling rapidly synchronize drinking activity with the opportunity to exercise during SVE.** Representative average waveforms of drinking activity for WT, *Vipr2$^{-/-}$*, *Vip$^{-/-}$* and *Vip$^{-/-}$Vipr2$^{-/-}$* mice. WT average waveform is plotted for the last 3 days of a 3-week SVE experiment (note that drinking activity onset precedes onset of wheel availability as WT mice free-run across window of SVE running wheel availability (also see **Fig. 1a and S1d**)). *Vipr2$^{-/-}$*, *Vip$^{-/-}$* and *Vip$^{-/-}$Vipr2$^{-/-}$* average waveforms are plotted for the entire 3 weeks of SVE. Note that, for all 3 genotypes with disrupted VIP-VPAC$_2$ signaling, the onset of drinking and wheel activity are well synchronized throughout SVE. (**d**) Double-plotted drinking activity examples (blue) from **Fig. 1a** for WT, *Vipr2$^{-/-}$*, *Vip$^{-/-}$* and *Vip$^{-/-}$Vipr2$^{-/-}$* mice during 3-week SVE experiments, shown without wheel-running overlaid, to aid visualization of timing of drinking. Shaded areas represent darkness. Red boxes indicate time of wheel availability during SVE.

(**e**) **Differential phase relationships between Zeitgebers and mice of different genotypes.** Phase plot showing the time of locomotor activity onset (mean± SEM) of each genotype plotted relative to Zeitgeber Time (ZT). For LD-DD1, ZT12 = time of lights off; for SVE-DD2, ZT12 = time of wheel unlock.

(**f**) **WT Mice stably entrain to longer durations of 24h period SVE.** Representative double-plotted actogram showing an additional example of a WT mouse stably entrained to a longer term 24h-period SVE protocol (~70 days in this example). Shaded areas represent darkness. Red boxes indicate time of wheel availability during SVE.

(**g**-**h**) Replotted bar charts showing from **Fig. 1g** and **Fig. 1h**, showing period (**g**) and rhythm strength (**h**) of wheel-running activity. Bars and error bars in panels show the mean± SEM for the whole dataset, as in **Fig. 1**, while the individual data point symbols show all data points at pre- and post-SVE, not just those that contributed to repeated measures statistical tests.

Pink-filled bars represent pre-SVE data, while red-filled bars represent post-SVE data.

In SFig1. statistical significance represented as: *,p<0.05.; **p<0.01; ***p<0.0001; ****p<0.00005.

Figure - S2

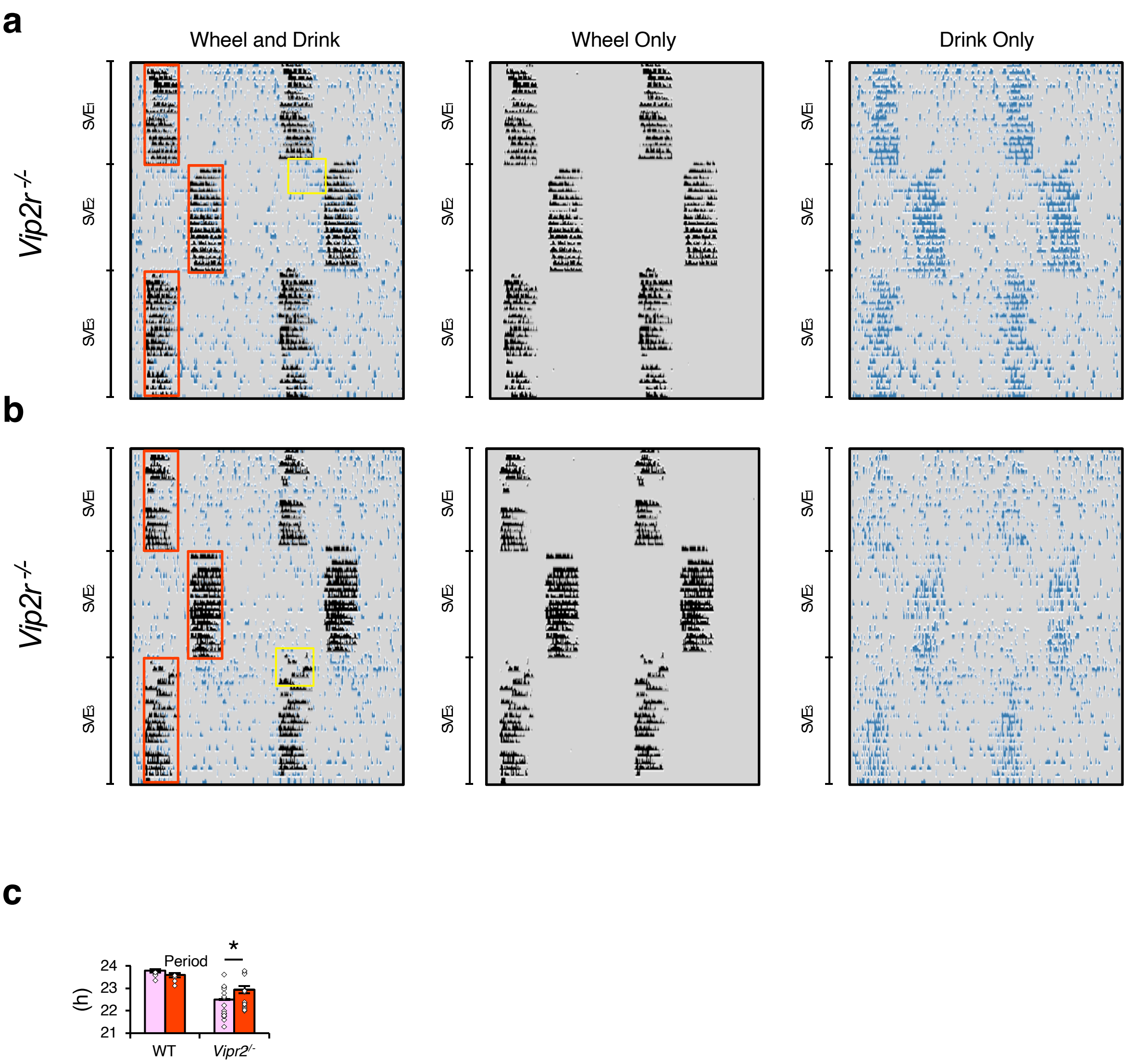

Figure S2.

(**a**-**b**) ***Vipr2**$^{-/-}$* **mice rapidly re-entrain to shifts in the phase of SVE but do exhibit transients**. Representative double-plotted actograms showing locomotor activity (black) and drinking activity (blue) for *Vipr2$^{-/-}$* mice (n=18) subjected to an 8h delay and 8h advance of a 6h/day SVE protocol. Shaded areas represent darkness. Red boxes indicate time of wheel availability during SVE. Yellow boxes highlight the occurrence of transient during re-entrainment after a shift in the phase of SVE. For the mouse in panel (**a**) transient cycles can be seen in drinking activity after the delay phase shift, and for the mouse in panel (**b**) transient cycles can be seen in wheel activity after the advance phase shift.

(**c**) Replotted bar chart from **Fig. 2b** showing period of wheel-running activity for mice in the 8-day SVE experiment. Bars and error bars show the mean± SEM for the whole dataset, as in **Fig. 2b**, while the individual data point symbols show all data points at pre- and post-SVE, not just those that contributed to repeated measures statistical tests.

Pre-SVE measures indicated by indicated by pink-filled bar and post-SVE by red-filled bar.

$^{*}p<0.05$

Figure - S3

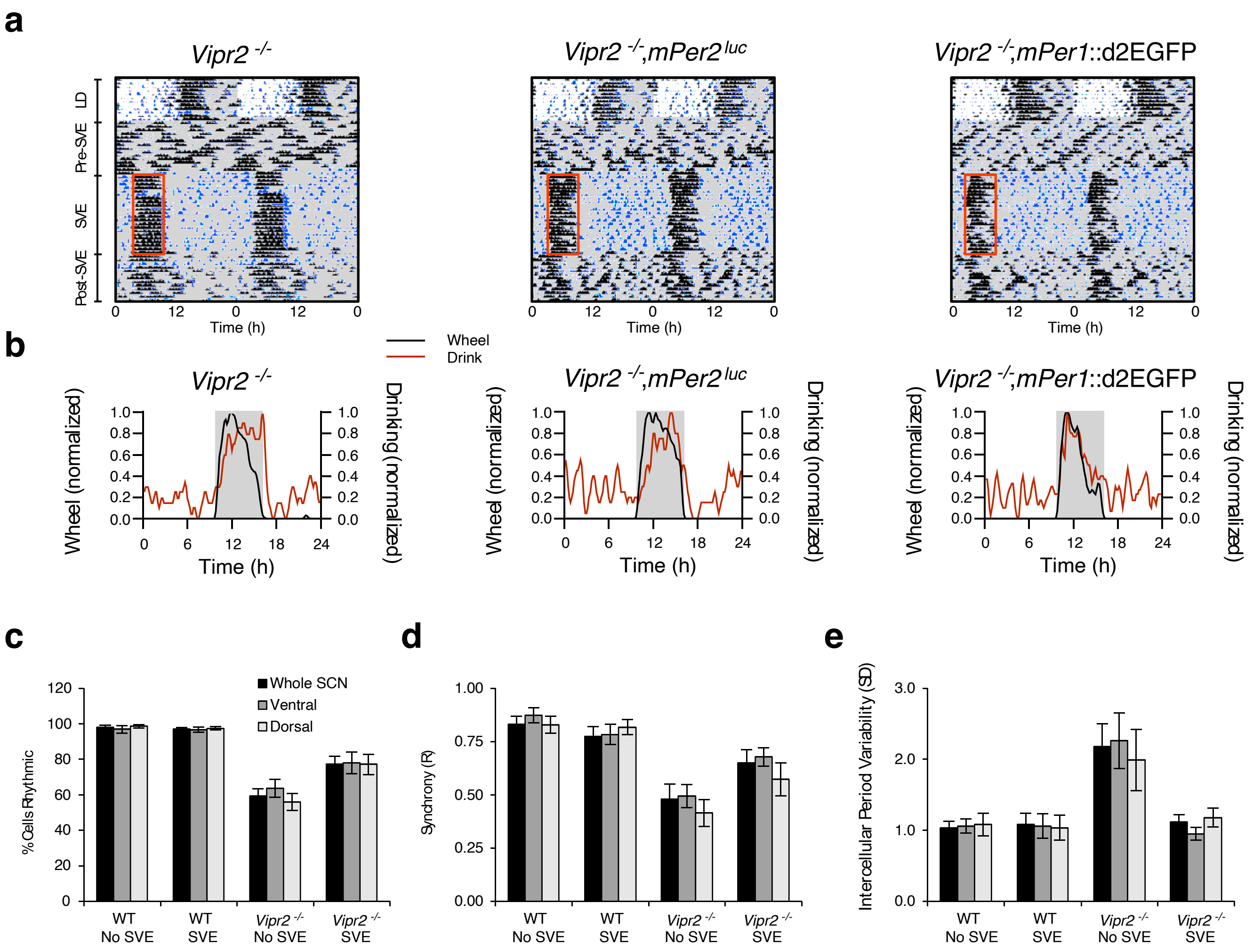

Figure S3.

(**a-b**) ***mPer2^{luc}* and *mPer1*::d2eGFP reporter backgrounds do not alter the responses of *Vipr2$^{-/-}$* mice to SVE.** (**a**) Representative double-plotted actograms showing locomotor activity (black) and drinking activity (blue) for *Vipr2 -/-* (no reporter), *Vipr2$^{-/-}$*,*mPer2^{luc}* and *Vipr2$^{-/-}$*,*mPer1::d2eGFP* mice (n=18, 17 and 6, respectively) undergoing the 3-week SVE protocol. Shaded areas represent darkness. Red boxes indicate time of wheel availability during SVE. (**b**) Representative average waveforms for the entire 3 weeks of SVE. Note that reporter background mice exhibit the same behavioral responses to 3-week SVE as non-reporter mice; disrupted/short period rhythms pre-SVE, rapid synchronization of activity with the opportunity to exercise during SVE, and the promotion of ~24h rhythms post-SVE.

(**c-e**) ***mPer1*::d2eGFP rhythm parameters did not differ between the dorsal and ventral regions of the SCN.** Bar charts of analyzed *mPer1*::d2eGFP rhythm parameters from **Fig. 3c**, showing whole SCN analysis (as shown in **Fig. 3c**, for comparison; black bars), as well as regional analyses of dorsal SCN (dark gray bars) and ventral SCN (light gray bars). No significant differences between dorsal and ventral SCN were detected in any rhythm parameters analyzed.

Figure-S4

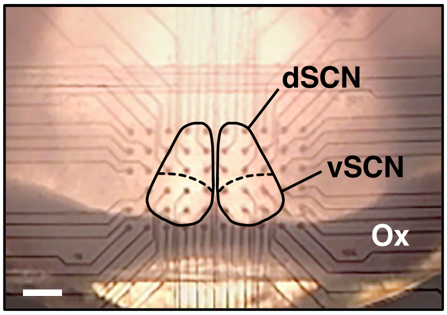

Figure S4.

SCN-MEA electrode alignment and anatomical subdivision of the SCN. Merged photomicrograph showing a typical SCN brain slice on the microelectrode array (MEA) with an MEA image and approximate SCN outline overlaid to aid visual assessment of the location of electrode terminals. dSCN and vSCN, dorsal and ventral parts of the SCN, respectively; Ox, optic chiasm. Scale bar = 200µm.

Figure - S5

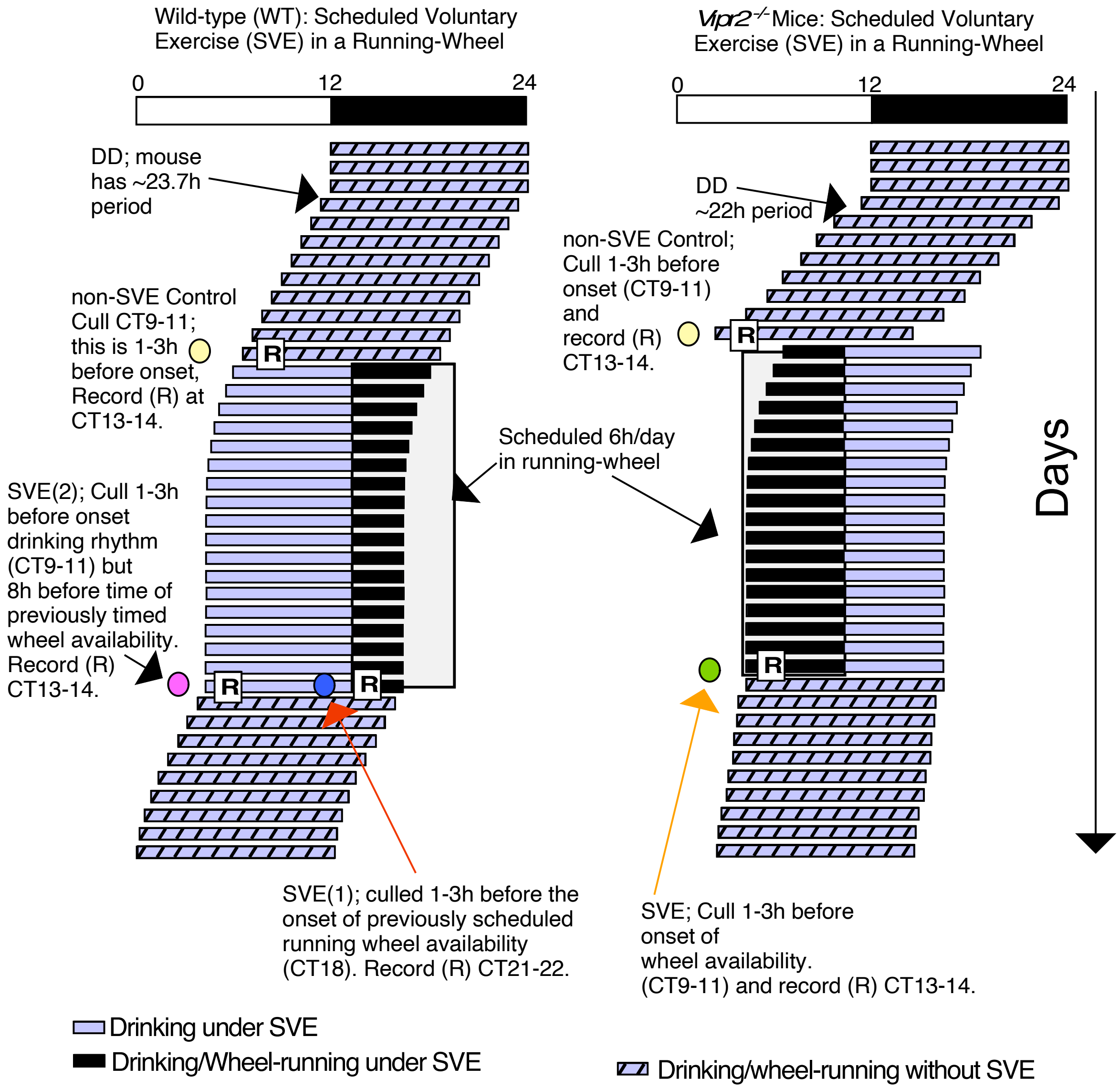

Figure S5.
Animals were individually housed in cages equipped with running-wheels as well as drinkometers. Initially mice were exposed to a 12h:12h light-dark cycle (LD) and then released to free-run in constant dark (DD). Non-SVE mice of WT and *Vipr2*$^{-/-}$ backgrounds were culled (yellow filled circles) during the subjective day at CT9-11 (or randomly from non-SVE control mice with no overt behavioral rhythms). Post-scheduled exercise *Vipr2*$^{-/-}$ mice were culled 1-3h prior to scheduled wheel availability (green filled circle), a time that also corresponded to 1-3h prior to activity onset as these mice maintain behavioral activity rhythms in phase with the opportunity to exercise. Since WT mice stably entrain ~8h advanced of the opportunity to exercise, post-scheduled exercise WT mice were culled at two different times to control for the time of exercising in the running wheel and endogenous behavioral (drinking) onset, separately. These two WT groups [SVE(1) and SVE(2), respectively], were culled 1-3h prior to the time of wheel availability (blue filled circle) and 1-3h prior to endogenous behavioral onset (purple filled circle). Both groups of WT SVE mice received the same SVE paradigm, only differing in the phase of cull time and subsequent recording phase on the MEA. Recordings (R) were initiated ~2-3h after cull. Horizontal unfilled/black bars at top show times of lights on/off during initial LD cycle. Shaded box shows time of wheel availability during SVE.

Table S1: Effects of Exercise on Spontaneous Multi-Unit Activity in the Dorsal and Ventral Subregions of the Suprachiasmatic Nuclei (SCN) of Neurochemically Intact (WT) and $VPAC_2$ Receptor Knockout (*Vipr2* $^{-/-}$) Mice.

**Subregion**	**Test**		**Significance**
Dorsal	One-way ANOVA		
	$F_{(4,271)}=3.08$		$p=0.017$
	Tukey HSD *Post-hoc*		
	Mean Difference	lql	
WT Con vs WT SVE(1)	0.04	0.09	*p*=1
WT Con vs WT SVE(2)	1.08	2.45	*p*=0.42
WT SVE(1) vs WT SVE(2)	1.12	2.62	*p*=0.35
Vipr2 $^{-/-}$ Con vs *Vipr2* $^{-/-}$ SVE	1.51	3.99	*p*=0.041
WT Con vs *Vipr2* $^{-/-}$ Con	0.63	1.51	P=0.8
Ventral	One-way ANOVA		
	$F_{(4,220)}=26.44$		$p<0.0001$
	Tukey HSD *Post-hoc*		
	Mean Difference	lql	
WT Con vs WT SVE(1)	3.96	6.16	*p*<0.0002
WT Con vs WT SVE(2)	2.13	3.32	*p*=0.14
WT SVE(1) vs WT SVE(2)	1.84	3.13	*p*=0.18
Vipr2 $^{-/-}$ Con vs *Vipr2* $^{-/-}$ SVE	0.8	1.35	*p*=0.88
WT Con vs *Vipr2* $^{-/-}$ Con	6.94	10.81	*p*<0.0001

Table S2: Effects of Exercise on Gabazine Evoked Changes in Multi-Unit Activity in the Dorsal and Ventral Subregions of the Suprachiasmatic Nuclei (SCN) of Neurochemically Intact (WT) and $VPAC_2$ Receptor Knockout (*Vipr2* $^{-/-}$) Mice.

**Subregion**	**Test**		**Significance**
Dorsal	One-way ANOVA		
	$F_{(4,205)}$=17.86		p<0.0001
	Tukey HSD *Post-hoc*		
	Mean Difference	Iql	
WT Con vs WT SVE(1)	6.19	8.97	p<0.0001
WT Con vs WT SVE(2)	3.64	5.05	p=0.0004
WT SVE(1) vs WT SVE(2)	2.54	3.56	p=0.091
Vipr2$^{-/-}$ Con vs *Vipr2*$^{-/-}$ SVE	1.18	1.58	p=0.8
WT Con vs *Vipr2*$^{-/-}$ Con	6.38	9.25	p<0.0001
Ventral	One-way ANOVA		
	$F_{(4,149)}$=8.3		p<0.0001
	Tukey HSD *Post-hoc*		
	Mean Difference	Iql	
WT Con vs WT SVE(1)	3.57	6.05	p=0.0003
WT Con vs WT SVE(2)	2.36	3.98	p=0.044
WT SVE(1) vs WT SVE(2)	1.2	2.25	p=0.51
Vipr2$^{-/-}$ Con vs *Vipr2*$^{-/-}$ SVE	0.03	0.04	p=1
WT Con vs *Vipr2*$^{-/-}$ Con	4.94	6.45	p<0.0001
