# Supplementary material for: Timed daily exercise remodels circadian rhythms in mice
Source: Commun Biol. 2021 Jun 18;4:761. doi: 10.1038/s42003-021-02239-2 (PMC8213798; doi:10.1038/s42003-021-02239-2)
Supplement: Supplementary file 3 — Description of Additional Supplementary Files [file 42003_2021_2239_MOESM3_ESM.pdf]

Description of Additional Supplementary Files

File name: Supplementary Data 1

Description: Summary data for main figures 1-7.
