# Supplementary material for: Timed daily exercise remodels circadian rhythms in mice
Source: Commun Biol. 2021 Jun 18;4:761. doi: 10.1038/s42003-021-02239-2 (PMC8213798; doi:10.1038/s42003-021-02239-2)
Supplement: Supplementary file 5 — Reporting Summary [file 42003_2021_2239_MOESM5_ESM.pdf]

nature research

Corresponding author(s): Hugh Piggins
Last updated by author(s): 2021 05 06

Reporting Summary

Nature Research wishes to improve the reproducibility of the work that we publish. This form provides structure for consistency and transparency in reporting. For further information on Nature Research policies, see our Editorial Policies and the Editorial Policy Checklist.

Statistics

For all statistical analyses, confirm that the following items are present in the figure legend, table legend, main text, or Methods section.

n/a	Confirmed	
☐	☒	The exact sample size (*n*) for each experimental group/condition, given as a discrete number and unit of measurement
☐	☒	A statement on whether measurements were taken from distinct samples or whether the same sample was measured repeatedly
☐	☒	The statistical test(s) used AND whether they are one- or two-sided<br>*Only common tests should be described solely by name; describe more complex techniques in the Methods section.*
☒	☐	A description of all covariates tested
☐	☒	A description of any assumptions or corrections, such as tests of normality and adjustment for multiple comparisons
☐	☒	A full description of the statistical parameters including central tendency (e.g. means) or other basic estimates (e.g. regression coefficient) AND variation (e.g. standard deviation) or associated estimates of uncertainty (e.g. confidence intervals)
☒	☐	For null hypothesis testing, the test statistic (e.g. *F*, *t*, *r*) with confidence intervals, effect sizes, degrees of freedom and *P* value noted<br>*Give P values as exact values whenever suitable.*
☒	☐	For Bayesian analysis, information on the choice of priors and Markov chain Monte Carlo settings
☒	☐	For hierarchical and complex designs, identification of the appropriate level for tests and full reporting of outcomes
☒	☐	Estimates of effect sizes (e.g. Cohen's *d*, Pearson's *r*), indicating how they were calculated

Our web collection on statistics for biologists contains articles on many of the points above.

Software and code

Policy information about availability of computer code

Data collection	Wheel-running and drinking activities were recorded using either Chronobiology Kit (Stanford Software Systems, Santa Cruz, CA., USA) or Phenomaster (TSE Systems, Bad Homburg, Germany) software. PMT-based bioluminescence recordings were made using a simple custom written event-counting script. Lumicycle-based bioluminescence recordings were collected using Lumicycle Collect Software (Actimetric, Evanston, IL., USA). Confocal images were acquired using Nikon C1 software (Nikon, Kingston-upon-Thames, UK). MEA data were acquired using MC_Rack software (Multichannel Systems GmbH, Reutingen, Germany).
Data analysis	Many data were analyzed using MS Excel or Kaleidagraph (Synergy Software, Reading Pa., USA). Behavioural periods were calculated pre- and post-SVE behaviour using Chi-squared periodogram analysis in Analyze9 (Stanford Software Systems) and Clocklab (Actimetrics). Luminescence and confocal images were analyzed using ImageJ (NIH, USA). Analysis of MEA data was done using Neuroexplorer (Nex Technologies, Madison, Ala., USA) and Spike 2 (Cambridge Electronic Design, Cambridge, UK). Statistical tests were performed using Systat 10 (SPSS, Chicago, IL., USA), MS Excel, Matlab (Mathworks, Natwick, MA., USA), EL Temps (Dr.A. Diez-Nogurea, Barcelona, Spain), Kaleidagraph (Synergy), and GraphPad online calculator: www.graphpad.com/quickcalcs/; Graphpad Software, San Diego, CA., USA).

For manuscripts utilizing custom algorithms or software that are central to the research but not yet described in published literature, software must be made available to editors and reviewers. We strongly encourage code deposition in a community repository (e.g. GitHub). See the Nature Research guidelines for submitting code & software for further information.

Data

Policy information about availability of data

All manuscripts must include a data availability statement. This statement should provide the following information, where applicable:

- Accession codes, unique identifiers, or web links for publicly available datasets
- A list of figures that have associated raw data
- A description of any restrictions on data availability

Datasets are available by request to the Lead Contact

Field-specific reporting

Please select the one below that is the best fit for your research. If you are not sure, read the appropriate sections before making your selection.

- [x] Life sciences
- [ ] Behavioural & social sciences
- [ ] Ecological, evolutionary & environmental sciences

For a reference copy of the document with all sections, see nature.com/documents/nr-reporting-summary-flat.pdf

Life sciences study design

All studies must disclose on these points even when the disclosure is negative.

Sample size	No sample size calculations were performed.
Data exclusions	No data were excluded from analyses for the experiments reported in this manuscript.
Replication	All experiments were completed as multiple independent experimental runs, with a minimum of 3 replicate independent experiments. Most experiments (behavior, MEA recordings, fluorescence imaging, bioluminescence recordings) were conducted as ongoing staggered experiments, with multiple repeated replicates.
Randomization	Mice were allocated into experimental groups at random.
Blinding	For behavioral experiments involving scheduled voluntary exercise (SVE) and controls, blinding during behavioral data collection and behavioral data analysis were not possible (one can see if the wheel moves or not during data collection; behavioral traces are distinctly different between SVE and control conditions and this is plainly visible during analysis of raw behavioral data). Blinding was not possible during experimental set up for MEA, fluorescence or bioluminescence recordings as the experimenter had to retrieve a mouse from from either an SVE or control cage. Where possible, data were blinded before the extraction of experimental parameters from collected data for all experiments (excluding behavior, see above).

Reporting for specific materials, systems and methods

We require information from authors about some types of materials, experimental systems and methods used in many studies. Here, indicate whether each material, system or method listed is relevant to your study. If you are not sure if a list item applies to your research, read the appropriate section before selecting a response.

Materials & experimental systems

n/a	Involved in the study	
☐	☒	Antibodies
☒	☐	Eukaryotic cell lines
☒	☐	Palaeontology and archaeology
☐	☒	Animals and other organisms
☒	☐	Human research participants
☒	☐	Clinical data
☒	☐	Dual use research of concern

Methods

n/a	Involved in the study	
☒	☐	ChIP-seq
☒	☐	Flow cytometry
☒	☐	MRI-based neuroimaging

Antibodies

Antibodies used	anti-VIP (1:2000; Enzo Life Sciences, Exeter, UK) and anti-VPAC2 (1:5000; Abcam, Cambridge, UK)
Validation	These antibodies are regularly used in the Piggins laboratory and have previously been validated in-house using relevant pre-absorption controls, as well as negative controls with the omission of primary or 2nd anti-bodies.

Animals and other organisms

Policy information about studies involving animals; ARRIVE guidelines recommended for reporting animal research

Laboratory animals	Adult (8-24 weeks old) male laboratory mice. C57Bl/6 (WT; Harlan, Blackthorn, UK); Vipr2-/- (originally derived from breeding stock from the late Prof. Harmar--Harmar et al., Cell v109, 2002; mPer2luc (Yoo et al., PNAS v101, 2004); Vipr2-/-,mPer2luc (Hughes et al. PlosOne e18926, 2011); mPer1::d2egfp (originally derived from Kuhlman et al., Neuroreport v11, 2000); Vipr2-/-,mPer1::d2egfp (Hughes et al., J. Neurochem v106, 2008); Vip-/- (Colwell et al. Am. J. Physiol. Reg. Int. v285, 2003); and Vip-/-Vipr2-/- (bred in-house at the University of Manchester by crossing Vip-/- and Vipr2-/- strains).
Wild animals	Study did not involve wild-animals.
Field-collected samples	Study did not involve field-collected samples.
Ethics oversight	All experiments were performed in accordance with the UK Animals (Scientific Procedures) Act of 1986 using procedures approved by The University of Manchester Ethics Panel.

Note that full information on the approval of the study protocol must also be provided in the manuscript.
